# “What do you know?”——knowledge among village doctors of lead poisoning in children in rural China

**DOI:** 10.1186/s12889-017-4895-2

**Published:** 2017-11-23

**Authors:** Ruixue Huang, Huacheng Ning, Carl R. Baum, Lei Chen, Allen Hsiao

**Affiliations:** 1 Department of Occupational and Environmental Health, Xiangya School of Public Health, Central South University, Changsha, China; 2 Pediatrics and of Emergency Medicine, Yale University School of Medicine, New Haven, USA; 3 Section of Emergency Medicine, Department of Pediatrics Faculty, Global Health Initiative, Yale University School of Medicine, New Haven, USA

**Keywords:** Children, China, Lead poisoning, Village doctors

## Abstract

**Background:**

This study evaluates the extent of village doctors’ knowledge of lead poisoning in children in rural China and assesses the characteristics associated with possessing accurate knowledge.

**Methods:**

A cross-sectional, questionnaire-based survey of 297 village doctors in Fenghuang County, Hunan Province, China was conducted. All village doctors were interviewed face-to-face using a “What do you know” test questionnaire focusing on prevention strategies and lead sources in rural children.

**Results:**

A total of 287 (96.6%) village doctors completed the survey in full. Most village doctors had an appropriate degree of general knowledge of lead poisoning; however, they had relatively poor knowledge of lead sources and prevention measures. Village doctors with an undergraduate level education scored an average of 2.7 points higher than those who had a junior college level education (*p* = 0.033). Village doctors with an annual income ≤ 10,000 RMB yuan scored 1.03 points lower than those whose income was >10,001 RMB yuan. Ethnic Han village doctors scored 1.12 points higher, on average, than ethnic Tujia village doctors (*p* = 0.027).

**Conclusions:**

This study identified important gaps in knowledge concerning lead poisoning in children among a rural population of village doctors. There is a clear need for multifaceted interventions that target village doctors to improve their knowledge regarding lead poisoning in children. The “What do you know” questionnaire is a new tool to evaluate lead poisoning knowledge and education projects.

## Background

Lead is a heavy metal that is widely distributed throμghout the environment [1]. Children are particularly vulnerable to lead poisoning because their bodies are in an ongoing state of growth and development [2–5]. Most experts believe that there is no safe blood lead level (BLL) in children [6]. Even very low levels of lead exposure can affect nearly every system in children’s bodies [7, 8]. Millions of children are exposed to lead with a significant risk of damage to the brain and nervous system, resulting in impaired growth and learning/behavior problems including diminished IQ, hearing and speech problems, and criminal behavior [9, 10]. Lead is a heavy metal that is widely distributed throughout the environment. Children are particularly vulnerable to lead poisoning because their bodies are in an ongoing state of growth and development. Currently, Millions of children globally are exposed to lead with a significant risk of damage to the brain and nervous system, resulting in impaired growth and learning/behavior problems including diminished IQ, hearing and speech problems, and criminal behavior. In USA, in 1970s, preschool children were screened by the Centers for Disease Control and Prevention(CDC) to show that the median blood lead level was 15μg/dL and approximately 90% of them with the level beyond 10μg/dL. Based on this data, government of USA took scaled actions to decrease the level and in 2002, the level had dramatically declined to 1.9μg/dL[11]. Based on this, some experts recommend that the screening level should be changed to as low as 2μg/dL, however, CDC didn’t intent to take this because they consider that in current time it was very hard to find effective ways to lower the lever under 10μg/dL, and children couldn’t simply be divided as beyond or below 10μg/dL group, as well as the evidence of low lead exposure effects to children weren’t sufficient till now. in China, a diagnosis and therapy of lead poisoning in children guideline released by Health and Family Planning Commission in 2005 showed that the blood lead level over 10μg/dL could be considered as high blood lead level, only when the blood lead level over 20μg/dL could be considered as lead poisoning and should be treated[12]. Althoμgh the argument about lead exposure is existing, currently, more and more experts believe that there is no safe BLL in children. Even very low levels of lead exposure can affect nearly every system in children’s bodies. It has been reported that even low dose lead exposure for children are associated with development delay, sluggishness, fatigue, wight loss, irritability, learning difficulties, anemia, lower IQ, aggressive behavior[13].

It has been identified the sources of lead exposure in USA were mainly lead products and old houses painted with lead-based paints, which account for nearly 70% of elevated blood lead levels in children [14], followed by gasoline-polluted soil, dust. Some other sources include lead-contaminated foods and beverages, however, on the contrast, in China, the major source of lead exposure for children is the lead-contaminated soil, dust and water with the rapid development of industrialization over past decades. A survey conducted between 1990 and 2012 in China reported that the median BLLs in children aged 0 (newborns), 0–3, 3–7 and 7–18 years were 74.9, 46.4, 57.6 μg/L and 55.6 μg/L, respectively [15, 16]. With increased industrialization in China, lead poisoning in rural children has become an important public health concern [17]. According to reports published in 2011, the average blood lead concentration in 228 children in Huaining County, Anhui Province, was > 100 μg/L. In Jiyuan County, Henan Province, 1008 children residing near lead smelters had blood lead concentrations exceeding 250 μg/L. In August 2009, 851 children in Fengxiang County, Shaanxi Province, were diagnosed with lead poisoning because of waste discharge from a local smelter and more than 170 children were hospitalized. In the same period, 1354 children residing around the Wugang Manganese smelting plant in Hunan Province had blood lead concentrations > 100 μg/L [18]. In comparison, blood lead concentrations above 5 μg/L are considered very concerning in the United States, and children with levels > 45 μg/L are typically hospitalized for intensive treatment.

Many rural residents have chosen to move to cities for work, leaving their children in the care of grandparents or older siblings who may not have adequate understanding of lead poisoning risks [19, 20]. Given the widespread use of lead, these children may be at increased risk of lead exposure in rural China. It is crucial that village doctors, who provide primary and preventive care for rural residents in almost every village in China, are aware of the sources of, pathways of exposure to, and measures to reduce the risk of, lead poisoning in children. There are roughly 14 million village doctors in China that service a rural population of 0.7 billion [21]. Village doctors are considered as the gatekeepers of children’s health in rural areas. With few or no other healthcare providers available, they represent the front line for treating lead poisoning. The prevention and treatment of lead poisoning in children in rural China depends largely on the medical care provided by village doctors. These doctors are also the primary resource from whom parents and caregivers obtain information on lead poisoning [22, 23]. Lack of awareness is an obstacle to preventing lead poisoning in children. Greater awareness would help to reduce childhood lead poisoning because the work of village doctors could be publicized and promoted.

To date, lead poisoning interventions for children have focused on educating parents on how to reduce the risk of exposure [24–27]. No studies that assess village doctors’ baseline knowledge of childhood lead poisoning in rural areas are available. This study was designed to examine the level of lead poisoning knowledge among village doctors; determine demographic characteristics and knowledge status associated with lead poisoning in children discuss possible gaps between real-world village doctors’ knowledge and the ideal situation; and improve evidence-based interventions to enhance health outcomes and prevent lead poisoning in children in rural China.

## Methods

### Study design, setting, and participants

This study was conducted from May to July 2017 at Fenghuang County, Xiangxi Tujia and Miao Autonomous Prefecture, Hunan Province, China [28].

**Fig. 1:**
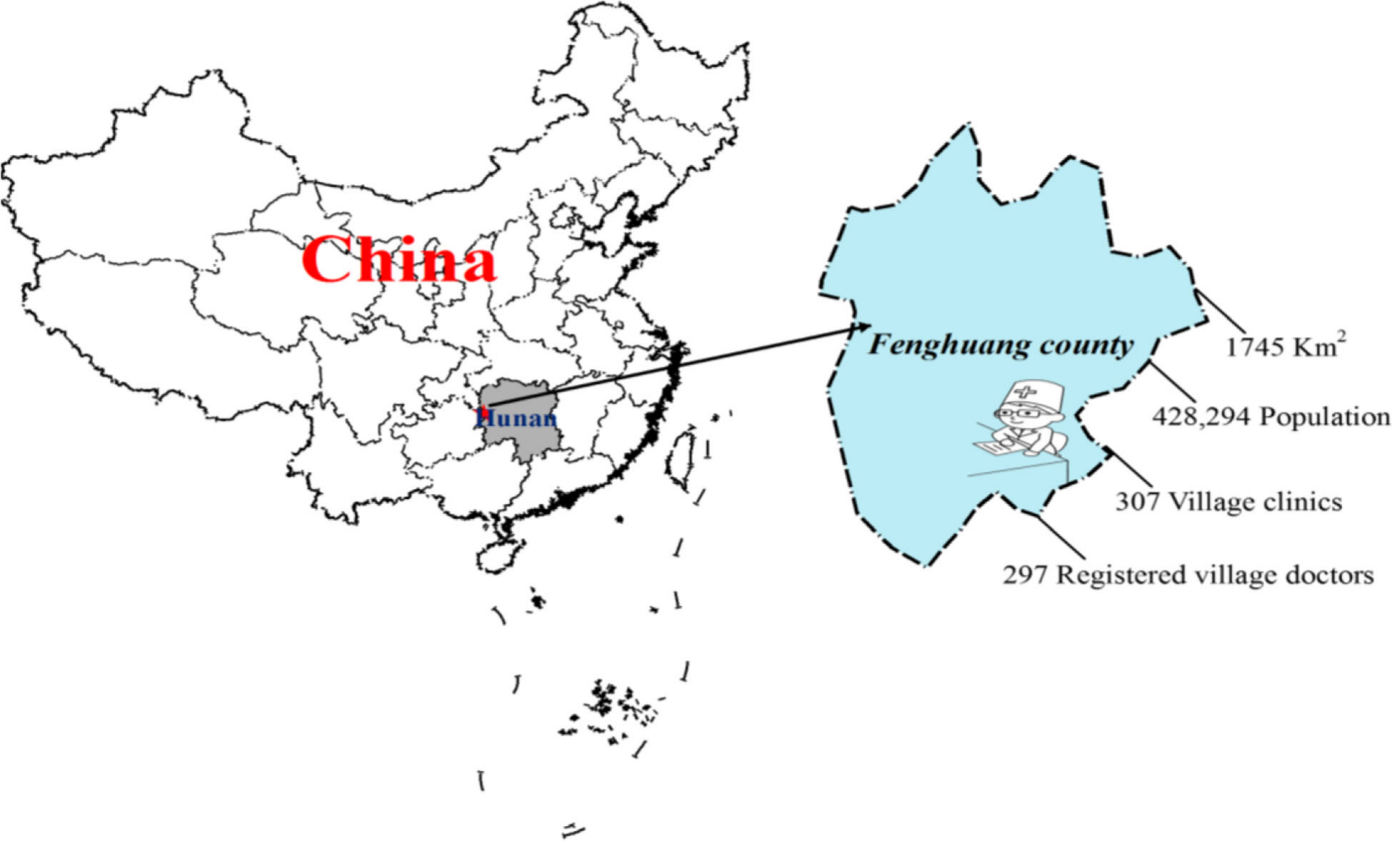
Location of Fenghuang county in Hunan Province, China

Xiangxi Tujia and Miao Autonomous Prefecture consists of seven counties: Baojing, Fenghuang, Guzhang, Huayuan, Longshan, Luxi, and Yongshun. Twenty-five nationalities are represented; of a total population of 2,480,000, 66.6% are ethnic minorities, including 860,000 Tujia and 790,000 Miao. Fenghuang County is located on the western margin of the Hunan province and the southern Xiangxi, and is immediately adjacent to the eastern edge of Guizhou Province. The county is bordered to the north by Huayuan County and Jishou City, to the east by Luxi County, to the southeast by Mayang County, and to the southwest and the west, respectively, by Tongren City, Bijiang District and Songtao County, Guizhou (Fig. 1). Fenghuang County covers 1745 km^2^ and had a registered population of 428,294 in 2015. Population statistics from a government population census are available at: http://www.stats.gov.cn/.

Fenghuang County is an ideal study location for several reasons. First, Fenghuang County is rich in mineral resources including lead, mercury and cadmium, and is known as the “capital of non-ferrous lead”. Fenghuang County contains 32 mineral deposits with an exploitation history of more than 1500 years. Children living in Fenghuang County have an increased exposure to heavy metals from the air, soil, and water. Children are especially vulnerable to such exposure because their bodies are still developing. Fenghuang County has 340 villages and is one of China’s poorest and most populous provinces, with more than half the population engaged in agriculture and living in rural or mountainous areas. A rural population of approximately 300,000 is served by only 297 village doctors. Village doctors are considered as the “gatekeepers” of rural residents’ health, and village doctors’ knowledge is critical to prevent lead poisoning in children. This study was supported by the Chia Fellowship of Yale-China Association, and by planned interventions to improve health outcomes for local, vulnerable and underserved populations, especially children. Fenghuang County has a total of 307 village clinics, with 297 registered village doctors available for local residents. All registered village doctors were recruited into this study. Local administrators provided a list of all village doctors’ names and telephone numbers. Surveys were read aloud by the interviewer and typically took between 10 and 25 min to complete. Study participants received $5. The sample size was calculated to provide sufficient power for post-intervention analyses, with an assumed nonresponse rate of 5%.

First, in order to develop this investigation scale, we searched the literatures regarding lead poisoning in children and the education training system of village doctors over past decades, as well as reference the USA and Chinese laws and guideline regarding lead poisoning diagnosis and treatment, the education training system of village doctors and assessed existing measures and validated scales, including the Chicago Lead Knowledge Test (CLKT). we also spoken with a few Health department officials and local village doctors and local government doctors regarding the acknowledge of lead poisoning in children. The interview contents included do you think it was necessary to educate village doctors about lead poisoning? Do you know some damages about lead exposure for children? Do you know how to block the exposure routes for children? Based on the previous work, we preliminarily developed relative nearly 20 items and then, Yale-China Chia fellows, Yale University professors and doctors, Xiangya School of Public Health professors, and the Fenghuang local village doctoral candidate co-improved the questionnaire. After discussing about the items, we added some items and deleted some items that we think the finally 28 items can be mostly reflect the Chinese rural status. For instance, the item about some traditional popular prescription such as “hongdan”, “zhangdan”, and “huangdan” do not include lead and safety for children has the Chinese rural characteristics since in rural areas, children with epilepsy are usually be treated with lead compounds by some village doctors, of which think this is a traditional and effective way, even the famous ancient book named The Compendium of Materia Medica had listed lead compounds to be the magic drug to treat epilepsy[29]. Based on the screening results, the knowledge survey was conducted using a 28-question test to determine village doctors’ knowledge of lead poisoning in children. The test, called “What do you know? A Chinese lead knowledge test”, includes 6 questions related to general information about lead, 4 about the clinical symptoms of lead poisoning in children, 11 about lead exposure risk sources, 3 about prevention strategies, and 4 about nutrition (Table 1). Self-reported demographic data, including age, gender, race, region, and zip code, were also collected. Possible test scores ranged from 0 to 28, with 28 being the highest possible score. A test score was derived for each respondent; correct responses were scored as 1, and incorrect or “I don’t know” responses were scored as 0. The frequency of correct, incorrect, and “I don’t know” responses were tabulated for each question and sorted into the “General knowledge,” “Exposures” “Prevention measures” and “Nutrition” categories of the survey. One-way analysis of variance (ANOVA) was used to determine associations between individual variables and test scores. An alpha level of 0.05 was used throughout.

**Table 1:** What do you know? A Chinese lead knowledge test

Questions	Rright key	right(%)	wrong(%)	don't know(%)
General knowledge				
1. Lead poisoning can be prevented. The key is to keep children from coming in contact with lead	True	23	23	54
2. No safe blood lead level in children has been identified. Lead exposure can affect nearly every system in the body,	True	12	21	67
3. Lead exposure often occurs in children with no obvious symptoms	True	66	23	11
4. Even low lead level in the body can affect a child’s IQ	True	26	47	27
5. Lead smelters, a battery recycling plant and other industries such as paintingare likely to dismiss lead to environment	True	88	12	
6. Children between the ages of 0 and 6 years old are the main victims of lead poisoning,	True	38	18	44
Exposure				
7. Lead paint is only found in newer decorated houses than in older houses	True	87	5	8
8. Using lead-containing glazed pottery for cooking in a short time would not increase the risk of lead poisoning in children	False	20	76	4
9. Furniture refinishing frequently can increase a child’s exposure to lead	True	88	2	10
10. Children who is usually putting their fingers into the month is easy to get lead poisoned	True	67	8	25
11. One way for the lead-containing dust is coming from some industries associated with utility lead	True	92	7	1
12. Parents who smoke in the house can increase the risk of lead poisoning in children	True	32	23	45
13. Parents who work with lead at their jobs can bring lead home on their hair, shin and clothes	True	38	43	19
14. Lead can be transferred to the fetus	True	15	56	29
15. Lead can be transferred to the brain and damage the child’s ability to learn	True	55	16	29
16. Some traditional popular prescription such as “hongdan”, “zhangdan”, and “huangdan” do not include lead and safety for children	False	9	79	12
17. Environment contamination with lead is the most widespread source of lead exposure for rural children	True	74	12	14
18. Using tin pots to cook or drink is another pathway for children to exposure lead	True	44	24	32
19. Lead poisoning in children may produce some symptoms like hard to pay attention and learn, causing behavior problems and the growth and development slow down	True	45	12	43
20. Toys and toy jewelry are also the risk factors for children exposure lead	True	39	58	3
21. Soil and tape water are the risk factors for children exposure lead	True	27	38	35
Prevention measures				
22. Teaching children washing their hands usually is good for preventing lead poisoning	True	82	10	8
23. If boiling the tap water lead can be removed	False	4	87	9
24. If the blood lead level is below 100μg/L, it doesn’t need to treat. Lead can leave the body as children grow up	False	6	91	3
Nutrition				
25. A small amount of lead is healthy for body because it can stimulate the immune system	False	12	85	3
26. Look for foods with calcium, iron, and vitamin D. these foods can help keep lead out of the body	True	78	22	0
27. Fresh fruit is healthy for children avoiding lead poisoning	True	79	21	
28. A diet with enough protein helps prevent lead poisoning in children	True	81	6	13

## Results

### “What do you know?” test characteristics

#### Content validity

Survey questions were developed collaboratively with Yale-China Association Chia fellows, Yale University professors, Yale New Haven Hospital pediatric doctors, and village doctors in Hunan Province. Based on feedback and the CLKT, additional information was included to reflect conditions in China, for example the association between lead and popular traditional prescriptions, such as “hongdan”, “zhangdan”, and “huangdan”. Furthermore, some items were removed, such as those pertaining to the association between lead and old houses, because in the study area most old houses were made of wood and never painted.

#### Test-retest reliability

Thirty-five doctors working at Xiangya affiliated hospital completed the survey twice. No more than 5 days elapsed between tests (range: 3–5 days). Calculated results for the percent agreement between two tests reflection to individual questions was 90–100%. The Pearson product-moment correlation for test scores was 0.96.

### Socio-demographic characteristics of the participants

A total of 297 village doctors participated in the survey, of whom 287 (96.6%) completed the questionnaire in full. Most respondents were over 50 years of age (Table 2). The mean age of the participants was 51.2 years (range: 28–72 years).

**Table 2:** Socio-demographic characteristics of the village doctors

Socio-demographic characteristics	Number (n)	Percentage (%)
Gender		
Male	206	71.7
Female	81	28.3
Age(year)		
≤ 35	37	12.8
36–49	54	18.8
≥ 50	196	68.4
Education level		
Junior and high school	76	26.4
Junior college	167	58.1
Undergraduate	44	15.5
Annual income(RMB yuan)		
≤ 10,000	89	31
10,001–15,000	149	51.9
≥ 15,001	49	17.1
ethnicity		
Han	26	9
Tujia	109	37.9
Miao	152	53.1
Marital status		
Married	207	72.1
Single	68	23.6
Widowed	12	4.3
Village doctors work experience		
< 2 years	23	8
2–10 years	185	64.4
> 10 years	79	27.6

### Knowledge of lead poisoning in children

As shown in Table 1, the mean score on the “What do you know?” test was 9.8 (SD 5.6). Each item indexed a certain aspect of lead knowledge among village doctors. The majority of village doctors responded correctly to survey items such as “Lead smelters, a battery recycling plant and other industrial activities, such as painting, are likely to discharge lead to the environment”, “Lead paint is only found in newer houses, not in older houses”, “Furniture refinishing can frequently increase a child’s exposure to lead” and “Look for foods with calcium, iron, and vitamin D. These foods can help keep lead out of the body”. In contrast, the majority of the village doctors responded incorrectly to questions such as “Boiling tap water can remove lead”, “If the blood lead level is below 100 ug/L, treatment is not required. Lead can leave the body as children grow up”, and “Some traditional popular prescriptions, such as “hongdan”, “zhangdan”, and “huangdan” do not include lead and are safe for children”. Generally, village doctors demonstrated good general knowledge and understanding of nutrition concerns, but more limited knowledge regarding lead exposure routes and preventive measures.

### Association between socio-demographic characteristics and “what do you know” scores

Table 3 presents the results of the one-way ANOVA analysis of “What do you know” test scores and socio-demographic characteristics. Village doctors with an undergraduate level education scored an average of 2.7 points higher than those with a junior college level education (*p* = 0.033). Furthermore, village doctors with an annual income ≤ 10,000 RMB yuan scored 1.03 points lower than those with an annual income > 10,000 RMB yuan. Ethnic Han village doctors scored, on average, 1.12 points higher than ethnic Tujia village doctors (*p* = 0.027).

**Table 3:** One-way ANOVA of “What do you know?” test scores

Independent variable	Difference	95% confidence limits	*p*-value
Age (years)			
≤ 35 vs. 36–49	0.28	(−0.36, 0.95)	0.16
36–49 vs. ≥ 50	0.47	(−1.43, 2.2)	0.55
Sex			
Male vs. female	1.7	(−0.5,4.1)	0.23
Education			
Junior college vs. undergraduate	−2.7	(−4.4, −0.2)	0.033^a^
Annual income (RMB yuan)			
≤ 10,000 vs. 10,001–15,000	−1.03	(−1.7, −2.2)	0.048^a^
10,001–15,000 vs. > 15,001	0.37	(−0.16, 0.5)	0.29
Ethnicity			
Han vs. Tujia	1.12	(−0.7,-3.4)	0.027^a^
Tujia vs. Miao	1.08	(−0.7,2.4)	0.67
Marital status			
Married vs. single	0.09	(−2.7,-1.1)	0.32

^a^indicates significance at the .05 level

## Discussion

Many developed counties, including the United States, have established policies and systems to prevent and control lead exposure in children, which have been proved to be effective in decreasing BLLs in children [30]. Over the past four decades, the BLLs of children in the USA have decreased dramatically following the introduction of strong measures and policies, including eliminating lead from gasoline and paint, banning lead solder in food cans, and the Lead Contamination Control Act of 1988. From 1976 to 1980, almost 85% of US children aged 1–5 years had a BLL > 10 μg/dL; by 1988–1991, this had decreased to only 5% of children [31]. Subsequently, government policy focused on prevention and control of low and very low BLLs. In low- and mid-income countries, such as China, India and some African countries, particularly those that have experienced rapid development and industrialization, lead poisoning in children is an ongoing challenge that must be addressed [32, 33].

Due to rapid economic development and industrialization, The main source lead pollution in environment in China is coming from lead smelting industry. In 1973, the first version of Lead Smelting Industry Pollution Emission Standards were launched by National Commission of Environmental Protection; in 1985 Emission standard for heavy non-ferrous metal industry pollutants was released and in 2007, A policy was founded that lead smelting mills cannot be built in cities or in suburbs, and in 2000, the production of leaded gasoline has been banned, and its sale has been banned as well by the National Development and Reform Commission, and in 2010 the Lead industry pollution emission standards were released. Although Chinese government take their attention intending to decrease the lead environment pollution, the pollution of lead still remains a scared issue. One is due to that more and more private factories select to move their factories from cities to rural settings in order to escape surveillance from the government or laws, another is due to the Chinese government continue to focus their attention on the economic development whereas disregards for environmental protection. For instance, some local government allow lead pollution industries such as battery factory or lead, zinc mining company to be existing in their local rural areas in order to pursuit the higher number of gross domestic product(GDP) values. Lead industries which are under protection of some local government may discharge pollutions and poisons into the environment including the air,water and soil. The ways to escape monitoring are various such as discharging during the intervals between monitoring or discharging at night or festival days. This status makes the lead poisoning in rural children become a serious public health concern. Some riots were happened in some rural areas because the children’s parents found their children’s blood lead level are very high. In the lead poisoning case of Shaanxi Province, the smelter accounted for 17% of the local government’s fiscal revenue in 2008.

Researchers with the Chinese Medical Association found that 65% of the 11,348 schoolchildren they tested had BLL concentrations [34] above the safe limit of 10 μg/dL, as set by the World Health Organization [35]. A meta-analysis showed that the lead poisoning rate among Chinese 0–1-year-old children was 28.1% (95% confidence interval [CI]: 21.6–34%) according to data published during 1990–2000, 5.3% (95% CI: 3.7–7%) during 2006–2012, and 9.6% in urban areas (95% CI: 7.1–12.1%) versus 23.8% in rural areas (95% CI: 6.7–40.9%). Most published literature reports discuss average BLLs, or mass incidents of lead poisoning, in children in rural China or near mining areas. Little has been reported regarding knowledge among doctors and parents regarding lead poisoning in children. It is necessary to identify possible sources of lead exposure for children; if parents, caregivers, and doctors have a good understanding of lead poisoning in children, they can help prevent lead exposure in this population.

To our knowledge, this survey is the first to evaluate village doctors’ awareness and knowledge of lead poisoning among children, and the first to apply a Chinese version of the “What do you know” survey to village doctors. We found that most village doctors had a general understanding of lead poisoning, but poor knowledge of preventive measures and lead sources. Village doctors with an undergraduate level education scored, on average, 2.7 points higher than those with a junior college level education (*p* = 0.033). This indicates that education plays an important role in increasing village doctors’ knowledge. Village doctors with an annual income below 10,000 RMB yuan scored 1.03 points lower than those whose income exceeded 10,001 RMB yuan. The income of village doctors is in the form of government payments, which are very limited, and payments from residents for herbs, medicines and medical services; the doctors’ incomes are very low compared to other occupations and village doctors thus may quit, taking up other occupations. On average, ethnic Han village doctors scored 1.12 points higher than ethnic Tujia village doctors (*p* = 0.027). Fenghuang County includes Tujia and Miao minorities, and cultural customs may affect the degree of understanding of lead poisoning in children. Lead and mercury are believed to have properties that cure some childhood disorders and allow people to live longer. Questions in the survey that address these beliefs were answered incorrectly by most of the village doctors. Fewer than 25 village doctors responded correctly to the question “Some traditional popular prescriptions, such as “hongdan”, “zhangdan”, and “huangdan” do not include lead and are safe for children”. “Hongdan”, “zhangdan”, and “huangdan” are lead-containing compounds that may adversely affect children’s health. In some rural areas of China, these compounds are believed to be able to cure epilepsy and skin diseases in children; some village doctors even sell them to local residents. The traditional use of lead powder for skin care has been shown to be a major contributor to elevated BLLs in children [36]. In 2016, Ying et al. reported that a 6-year old boy diagnosed with lead poisoning had an initial blood lead concentration of 63.6 μg/dL after ingesting a folk remedy for treating epilepsy [37]. The remedy, called Yu-Xian-Wan, contains a lead compound and was prescribed by a traditional healer. Of the 1082 incidents of drug-induced lead poisoning in China in the period 1981–2009, folk remedies accounted for 16.7% of the cases [38]. It should be noted that lead poisoning caused by traditional medicine is not limited to China, but occurs all over the world. Increased and improved health education, especially for local village doctors, caretakers, and private doctors could address this issue and thus prevent lead poisoning.

Increasing awareness about lead exposure sources and preventive measures among village doctors are crucial important and this is our next step to perform. First, we have contact our Health and Family Planning Commission of Hunan Province to get their assistance, each year, this commission have concentrated training in county towns for village doctors to enhance their medical knowledge. We can use this platform to disseminate the lead poisoning knowledge posts to them. Second, based on our previous study that not all the village doctors would intend to study in county town due to more time-wasted and more expense on the traffic and accommodation, we plan to take mobile phone texts and application to educate them since the mobilephone usage rate is pretty much high in rural areas. Now we are performing this protocol and applying for ethnic commission permission, we hope through education whatever by concentration training or mobile phone the knowledge regarding lead poisoning in children could be acknowledged by them. According to the investigation outcome, Some specific areas should be focused to education among village doctors. One is the general knowledge regarding no safe blood lead level in children has been identified; another is the lead exposure routes including parents smoking and using lead-containing glazed pottery for cooking and traditional popular prescription using, the third area is that boiling water can’t remove lead and it should treat children if the blood lead level is over 100μg/L. Finally, it should educate village doctors that a small amount of lead is also harmful for our health.

This study had several strengths, including a high response rate to the survey, and the use of a Chinese language version of the test instrument. A potential limitation is that only village doctors in Fenghuang County were surveyed. Although Fenghuang County was chosen, our sample may not have encompassed the characteristics of village doctors in all rural areas. Additionally, the survey only reported on the knowledge, and not the attitudes and practices, regarding prevention and control of lead poisoning in children among village doctors. However, our cross-sectional survey also has a few limitations, First, this survey was conducted in one Chinese county and caution is required when generalizing our results from this study to other different background. Second, followed by the first limitation, we recruited all the village doctors living in one county and the sample size didn’t be calculated based on the statistic method, future survey should be considered that the sample size recruiting should be based on a cluster randomized method or other method that can make better understanding the Chinese village doctors’ knowledge regarding lead poisoning in rural children.

## Conclusions

This study identifies an important lack of knowledge regarding lead poisoning in children among village doctors in rural China. Our findings underscore the need for interventions to improve knowledge among village residents and doctors regarding lead poisoning. Further studies are required to provide more information on the attitudes and practices of village doctors, and to more clearly delineate the role that village doctors play in setting expectations for use of traditional medicines by local residents.

## Abbreviations

BLL: Blood lead level
CDC: Centers for Disease Control and Prevention
CLKT: Chicago lead knowledge test
GDP: Gross domestic product
IQ: Intelligence quotient
RMB: Renminbi
SD: Standard deviation
